# Effects of a Collective Family-Based Mobile Health Intervention Called “SMARTFAMILY” on Promoting Physical Activity and Healthy Eating: Protocol for a Randomized Controlled Trial

**DOI:** 10.2196/20534

**Published:** 2020-11-11

**Authors:** Kathrin Wunsch, Tobias Eckert, Janis Fiedler, Laura Cleven, Christina Niermann, Harald Reiterer, Britta Renner, Alexander Woll

**Affiliations:** 1 Institute of Sports and Sports Science Karlsruhe Institute of Technology Karlsruhe Germany; 2 Department of Sports Science University of Konstanz Konstanz Germany; 3 Department of Computer and Information Science University of Konstanz Konstanz Germany; 4 Department of Psychology University of Konstanz Konstanz Germany

**Keywords:** mobile app, telemedicine, behavior change, health behavior, family, primary prevention, exercise, food and nutrition, randomized controlled trial, accelerometer, wearable electronic devices, social cognitive determinants, just-in-time adaptive intervention, digital intervention, mobile phone

## Abstract

**Background:**

Numerous smartphone apps are targeting physical activity and healthy eating, but empirical evidence on their effectiveness for initialization and maintenance of behavior change, especially in children and adolescents, is still limited.

**Objective:**

The aim of this study was to conceptualize a theory-based and evidence-based mHealth intervention called SMART*FAMILY* (SF) that targets physical activity and healthy eating in a collective family-based setting. Subsequently, the app will be refined and re-evaluated to analyze additional effects of just-in-time adaptive interventions (JITAIs) and gamification features.

**Methods:**

A smartphone app based on behavior change theories and behavior change techniques was developed and implemented and will be evaluated with family members individually and cooperatively (SF trial). Existing evidence and gained results were used to refine and will be used to re-evaluate the app (SF2.0 trial). Both trials are cluster randomized controlled trials with 3 measurement occasions. The intervention group uses the app for 3 consecutive weeks, whereas the control group receives no treatment. Baseline measurements (T_0_) and postintervention measurements (T_1_) include physical activity (ie, self-reported and accelerometry) and healthy eating measurements (ie, self-reported fruit and vegetable intake) as the primary outcomes. The secondary outcomes (ie, self-reported) are intrinsic motivation, behavior-specific self-efficacy, and the family health climate, complemented by an intentional measure in SF2.0. Four weeks following T_1_, a follow-up assessment (T_2_) is completed by the participants, consisting of all questionnaire items to assess the stability of the intervention effects. Mixed-method analysis of covariance will be used to calculate the primary intervention effects (ie, physical activity, fruit and vegetable intake) while controlling for covariates, including family health climate, behavior-specific self-efficacy, and intrinsic motivation.

**Results:**

This study is funded by the German Federal Ministry of Education and Research and ethically approved by the Karlsruhe Institute of Technology. For both trials, it is hypothesized that the apps will positively influence physical activity and healthy eating in the whole family. Furthermore, SF2.0 is expected to produce stronger effects (ie, higher effect sizes) compared to SF. SF app development and piloting are completed. Data acquisition for the SF trial is terminated and discontinued due to the COVID-19 pandemic. SF2.0 app development and piloting are completed, while data acquisition is ongoing. Participant recruitment for the SF 2.0 trial started in February 2020. The results for SF are expected to be published in mid-2021, and the results of SF2.0 are expected to be published in mid-2022.

**Conclusions:**

In this study, it is hypothesized that targeting the whole family will facilitate behavior change at the individual level and the family level, as the implemented strategies address changes in daily family life. Furthermore, subsequent app development (SF2.0) with supplementary addition of motivation-enhancing features and a JITAI approach is expected to enhance positive intervention effects.

**Trial Registration:**

German Clinical Trials Register DRKS00010415; https://tinyurl.com/yyo87yyu

**International Registered Report Identifier (IRRID):**

DERR1-10.2196/20534

## Introduction

### Background

A lack of physical activity, too much sedentary behavior (eg, extended screen time and nonactive media usage), and an unhealthy diet are serious concerns of modern societies. These behaviors increase the risk of health conditions across all ages [1-4]. Research has shown that many children and adolescents do not sufficiently engage in physical activity [5] and frequently make unhealthy food choices [6,7]. Approximately 81% of the children and adolescents (and 23% of the adults) in the world do not meet the recommendations on physical activity levels and healthy eating, for example, fruit and vegetable intake [8]. In this regard, a dose-response relationship was detected, with even slight increases in physical activity leading to physiological and psychological health benefits in adults [9-11] as well as in children and adolescents [12]. Longitudinal studies have shown that behavioral patterns in adolescence have low-to-moderate influence on physical activity patterns in adulthood [13-16]; therefore, there is a need for interventions targeting children to promote a sustainable and healthy lifestyle.

Health-related behaviors such as physical activity and healthy eating are embedded in social contexts such as the family context and are affected by social relations and ties [17]. Therefore, addressing behavioral changes embedded in daily family life might be a promising avenue for facilitating an individual’s behavior change. Family meals, for example, are often an important part of everyday life in families and there is accumulating evidence that this collective behavior is associated with a better overall diet quality and body mass index [18-20]. In a similar vein, there is some evidence that family-based physical activity is positively associated with individual physical activity levels [21]. It has been shown that supportive interactions within a family and shared values about health behavior affect children’s physical activity engagement [22] and eating behavior [23]. Moreover, results of intervention studies indicate that social support is significantly associated with continuation of exercise programs [24-28] as well as participation in weight-loss interventions [29-31].

Mobile health (mHealth) technologies are increasingly used as a delivery mode for health behavior change interventions throughout the lifespan. Specifically, smartphone-based apps offer a great promise for enhancing physical activity and healthy eating as well as for making health care more accessible and scalable, more cost-effective, and more equitable [32,33]. Reviews and meta-analyses support the view that app-based mobile interventions are effective and highly promising for changing physical activity [27,34] and nutrition behaviors [35]. Moreover, a recent systematic review of economic evaluations of mHealth solutions found a consistent overall reporting of positive economic outcomes (eg, increase in life-years gained, cost savings, cost-effectiveness) [36].

Reviews on mHealth interventions indicate that the strategies or the central “building blocks” of app-based interventions mainly encompass 4 behavior change technique clusters [37], namely, goal setting, feedback and self-monitoring, information, and social support provision, which coincide with successful conventional individual and group-based interventions [35,38,39]. Setting goals, monitoring behavior, receiving feedback, and reviewing relevant goals in the light of feedback are central to self-management and behavioral control, as specified by control theories [40,41] and health behavior theories [42-44]. However, since mobile interventions distinguish themselves by being interactive, adaptive, time-sensitive, and intraindividually dynamic, more dynamic concepts, including the timing of feedback or tailoring tasks and goals to individual progress and capacities as specified in persuasive technology and gamification approaches, might be essential ingredients of effective focused mobile interventions [35]. Moreover, mobile interventions can be delivered within a social system so that all members, for example, of a family, can simultaneously and collectively take part in an intervention and share their goals and progress. However, currently available apps for health promotion are almost exclusively tailored to the individual person [45]. Motivation for behavior performance is higher when the individual is embedded in a social system of mutual appreciation and importance (see self-determination theory [46,47]), which was successfully used in physical activity interventions by enhancing autonomous motivation and fulfilling the 3 basic psychological needs, that is, “autonomy,” “competence,” and “relatedness” [46]. As healthy or unhealthy behavioral patterns are developed and maintained in social contexts, embedding an mHealth intervention in a family-based setting and targeting all family members might be promising and corresponds to assumptions of family-as-systems approaches [48]. Families represent natural social systems characterized by supportive interactions and common shared values and should therefore be targeted as a whole to implement sustainable behavior change on the individual as well as the family level. Therefore, the described trials aim at developing a smartphone intervention app that targets the family as a social system of high relevance for its single members.

### Objective

The aim of the SMART*FAMILY* project, consisting of SMART*FAMILY* (SF) and SMART*FAMILY*2.0 (SF2.0) is to develop, refine, and evaluate an mHealth intervention aiming to improve physical activity levels and healthy eating at the individual and family level. The development of the app is based on behavior change theories, including self-determination theory and the use of behavior change techniques. Extending the previous research, the behavior of children *and* parents is targeted in order to induce family-based and individual-based behavior changes. In particular, family members are using the SF app individually and cooperatively. Furthermore, SF and SF2.0 aim to deliver context-dependent interventions and provide support during time periods when needed the most. The first version of the app (SF) will be refined, and motivational and gamification features as well as a just-in-time adaptive intervention (JITAI) approach will be added (SF2.0). The effectiveness of SF and SF2.0 will be evaluated through 2 cluster randomized controlled trials consisting of families (parents and their children).

## Methods

### Study Design

**Figure 1:**
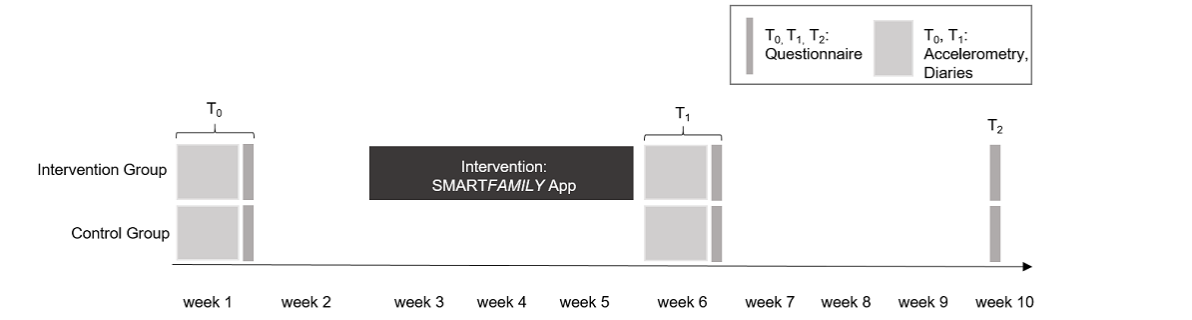
Detailed study design. T0: baseline; T1: after 3 weeks of intervention; T2: Four weeks after T1.

The studies are conducted and described according to the CONSORT-eHealth checklist [49], which can be found in Multimedia Appendix 1. The outline of the SF and SF2.0 trials is presented in Figure 1. In both trials, the assessment of outcomes is identical and is completed at baseline (T_0_) after the 3-week intervention (or no intervention) period (postintervention, T_1_), and 4 weeks after T_1_ (follow-up, T_2_). Each family (parents and children) is invited to the laboratory for an individual introductory session. Each family member receives an accelerometer for physical activity assessment and a daily paper-pencil–based diary assessing type, intensity, duration and joint activities as well as food intake (specifically fruit and vegetable intake) during the first assessment week (T_0_, Figure 1). At the end of week 1 (end of T_0_), they fill in a questionnaire (paper-pencil in SF, web-based questionnaire in SF2.0) and return all the materials to the laboratory. The completion of the questionnaire takes about 20 minutes. Both parents and children complete individual daily diaries and questionnaires. The data of the preintervention (baseline) assessment (T_0_) are analyzed and they serve as the basis for the weekly goal set-up in the intervention group during the following intervention period.

**Figure 2:**
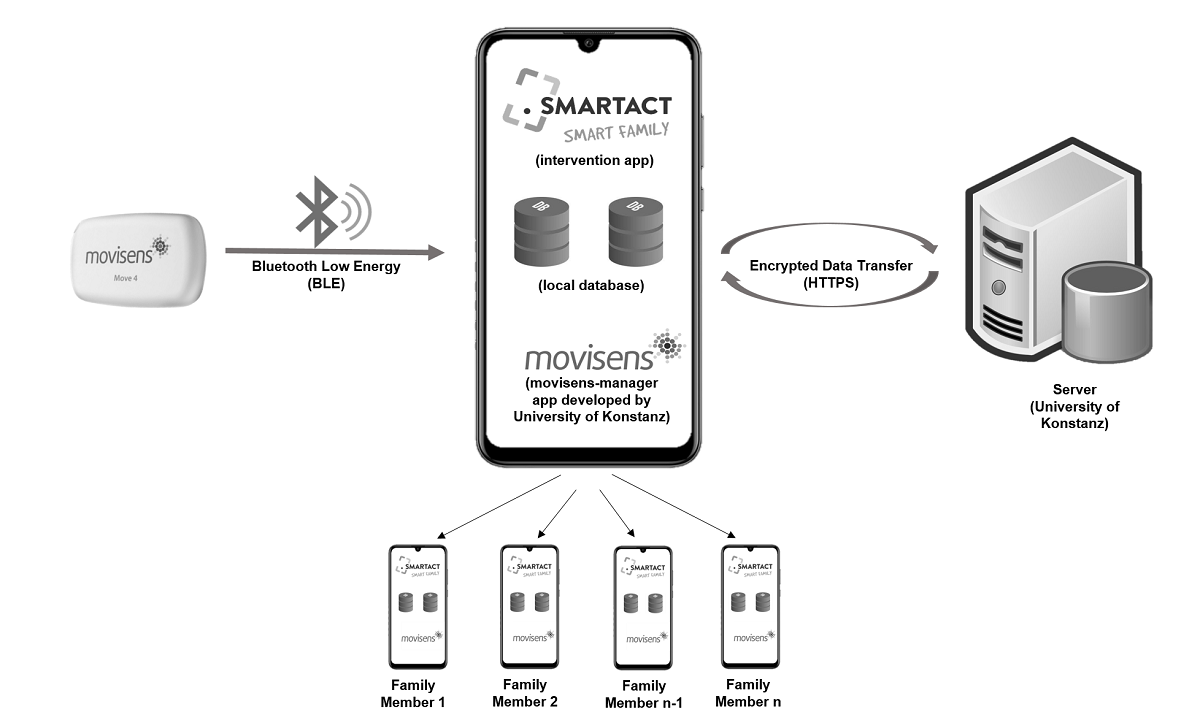
Detailed depiction of the operation principles of the SMARTFAMILY apps.

Participants allocated to the intervention group receive smartphones with the preinstalled SF app (SF or SF2.0). We provide all study participants with a study smartphone in order to control for device effects (screen size, Android version, etc) and ensure that the app is fully functioning. Carrying an additional device might pose an additional burden for participants and therefore, great care will be taken to explain the need for a study device in the introductory session. Moreover, previous studies within the SMARTACT consortium, comparing participants who could either use their own smartphone or were provided with a study smartphone to record their diet, showed no differences in terms of engagement [50]. Each smartphone (Samsung Galaxy A5 for SF, Nokia 5 for SF2.0) is connected with an accelerometer (Move 3 [SF] and Move 4 [SF2.0], Movisens GmbH) via Bluetooth low energy. Different from similar mHealth studies, the accelerometer used for the preintervention and postintervention (primary outcome) measurement is also used within the intervention period and provides data used by the app. Since participants monitor their physical activities (based on the data from the accelerometer) and goal progress through the app, an additional commercial device is not necessary for motivating the participants. Data are stored in a local database on the smartphone by using an additional app (Movisens Manager app) developed by the Human-Computer Interaction Group of the University of Konstanz as part of the SMARTACT project. The SF and SF2.0 apps are notified by the Movisens Manager app once new sensor data are available (ie, when the accelerometer is in reach of the Bluetooth low energy connection of the smartphone) and processes this data, thus creating 2 new entries in the database: the received data from the accelerometer and the aggregated value for that day (including accelerometer data and manual input by the participants). The aggregated data are then sent via encrypted HTTPS to the server in Konstanz. From there, data are sent back to all family members’ smartphones, so that individual and collective goal progress can be monitored. The control group does not receive any intervention. Please see Figure 2 for a depiction of the operation principles.

When starting the intervention, all families of the intervention group are asked to set a collective weekly goal for physical activities, which they want to achieve as a family within 1 week, that is, total steps and total time spent on physical activity per family. In addition, collective family goals are set for activities within the family and healthy eating, ie, the amount of fruit and vegetable intake per family and the number of family meals. It is important to note that every single family member is contributing to the collective family goal through his or her own behavior. In contrast to the set goals related to common recommendations such as 10,000 steps a day or 5 servings of fruits and vegetables per day for individuals, the family sets its own collective goals. In order to facilitate realistic goal-setting at the beginning of the intervention, the family members can rely on the analysis of their individual physical activity level and fruit and vegetable intake assessed during T_0_. The goal-setting process is repeated every week during the intervention period. The only goal-setting instruction given to the participants is the recommendation to set collective family goals for the coming week, which are slightly higher than their current cumulative performance. In SF2.0, an interactive goal-setting coach assists with this decision. This procedure involves usage of behavior change techniques, which will be presented in detail below.

After 3 weeks of intervention (or no intervention) period, a 1-week postintervention measurement (T_1_) with the same procedure as T_0_ starts. Four weeks after completion of T_1_, participants fill in the questionnaire for the last time (T_2_) and return them to the study team in a prepaid reply envelope. In SF2.0, participants can complete all questionnaires pseudonymized on the internet. Validated measures and scales are used (if available), which were adapted from paper-pencil versions for web-based versions to make it easier for participants to complete the surveys. Ethical approval was obtained and data protection was ensured. A closed survey design was used and the usability and technical functionality of the electronic questionnaire were tested. Participants receive analyses of their individual activity patterns for study participation in SF and SF2.0, whereas families in SF2.0 are additionally provided with a 40€ (US $46.8) online shopping voucher and an activity tracker for every child of the family in order to further facilitate physical activity maintenance.

### Eligibility Criteria and Ethical Approval

Families are eligible for inclusion if 1 parent or both parents and at least one child who is 10 years of age or older are living together in a common household. All siblings are invited to take part in the study. All participants have to be used to handle a smartphone and speak, read, and write German fluently. In order to ensure fairness, siblings who do not meet the age requirements in the inclusion criteria also receive the study materials, if deemed feasible.

Full ethical approval was obtained from the University of Konstanz (for the consortium SMARTACT) as well as from the Karlsruhe Institute of Technology (for SF and SF2.0). All participants, children, and legal guardians provide written informed consent prior to commencing the study by signing the informed consent form. Both trials are conducted in accordance with the Declaration of Helsinki.

### Randomization and Blinding

Both trials (SF and SF2.0) are cluster randomized controlled trials with 2 groups: (1) an intervention group receiving the SF or SF2.0 and (2) a nonintervention control group. Recruited families who provide informed consent are allocated to one of the 2 groups prior to recruitment by using a simple randomization procedure for cluster designs [51]. Although the intervention group participants are told about the mHealth nature of the study, control group participants are only told to take part in an epidemiologic assessment of physical activity levels, making it essential to wear the accelerometers 2 times for 1 week and to answer several questions over the course of 10 weeks in order to gain reliable and valid results.

### Participant Recruitment

**Figure 3:**
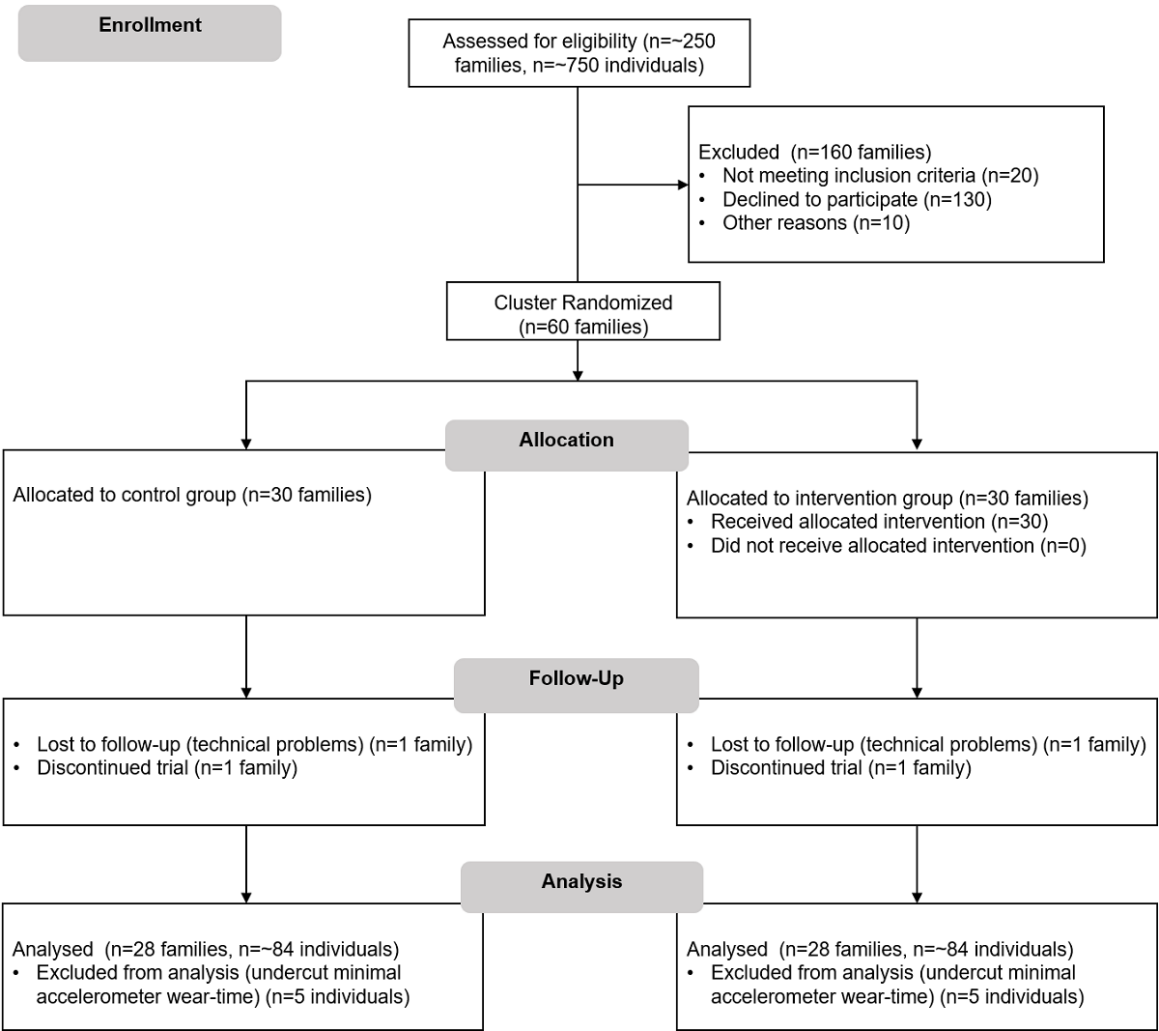
Planned participant flow and allocation pattern for the cluster randomized controlled trial for SMARTFAMILY and SMARTFAMILY2.0.

Participants are recruited in schools, school holiday programs, music schools, and sports clubs via personal communication, newspapers, and email distribution lists of the Karlsruhe Institute of Technology. Power analyses for analysis of covariance with 2 groups and 4 covariates using G*Power [52] yields a total of 52 families with approximately N=156 participants (assuming 3 family members), to find a small-to-medium effect (α=.05, 1-β=.80, Cohen *f*=0.25). In order to increase power and to compensate for potential dropouts, we aim for a total of 60 families per trial. Please see Figure 3 for the planned participant flow for SF and SF2.0.

### Development of SF Apps

Both SF and SF2.0 are developed as part of the SF project, which is part of the consortium project SMARTACT and its toolbox encompassing mobile interventions for promoting physical activity and healthy eating (see for example [53-57]). The multidisciplinary team includes professionals with expertise in sports and exercise science, nutrition, health psychology, neuroscience, economics, and human-computer interactions. SF and SF2.0 are devised in iterative processes, with input from target group members and experts as well as from previous SMARTACT project findings and behavioral theories. Programming of the apps is conducted by the Human-Computer Interaction Workgroup of the University of Konstanz as part of the SMARTACT project, which is responsible for all tasks related to computer science (ie, programming of apps, surveillance of data servers, etc). In general, all people with data access signed a confidentiality agreement. Both apps run on Android and use the SMARTACT Toolbox, which is conceptually developed by the SMARTACT consortium partners [53] and programmed within the SMARTMOBILITY project led by the Human-Computer Interaction Group of the University of Konstanz. The appearance and content of SF and SF2.0 are adapted and changed in iterative processes throughout the invention phases and pilot studies but not throughout a trial. Both apps are piloted for probing usability and feasibility through standardized interviews (SF) and questionnaires (SF2.0).

### Intervention: SF and SF2.0 Apps

Overall, SF and SF2.0 aim to enhance physical activity levels and healthy eating at the family level, including parents and children. The apps are designed to be implemented autonomously by participants. Both apps (SF and SF2.0) are entitled to fulfil criteria of high quality regarding theoretical and empirical foundation [38,46,48]. The inclusion of 10 (SF) and 13 (SF2.0) behavior change techniques doubles the amount of behavior change techniques found in “average” mHealth intervention apps.

### Features of the SF App

**Figure 4:**
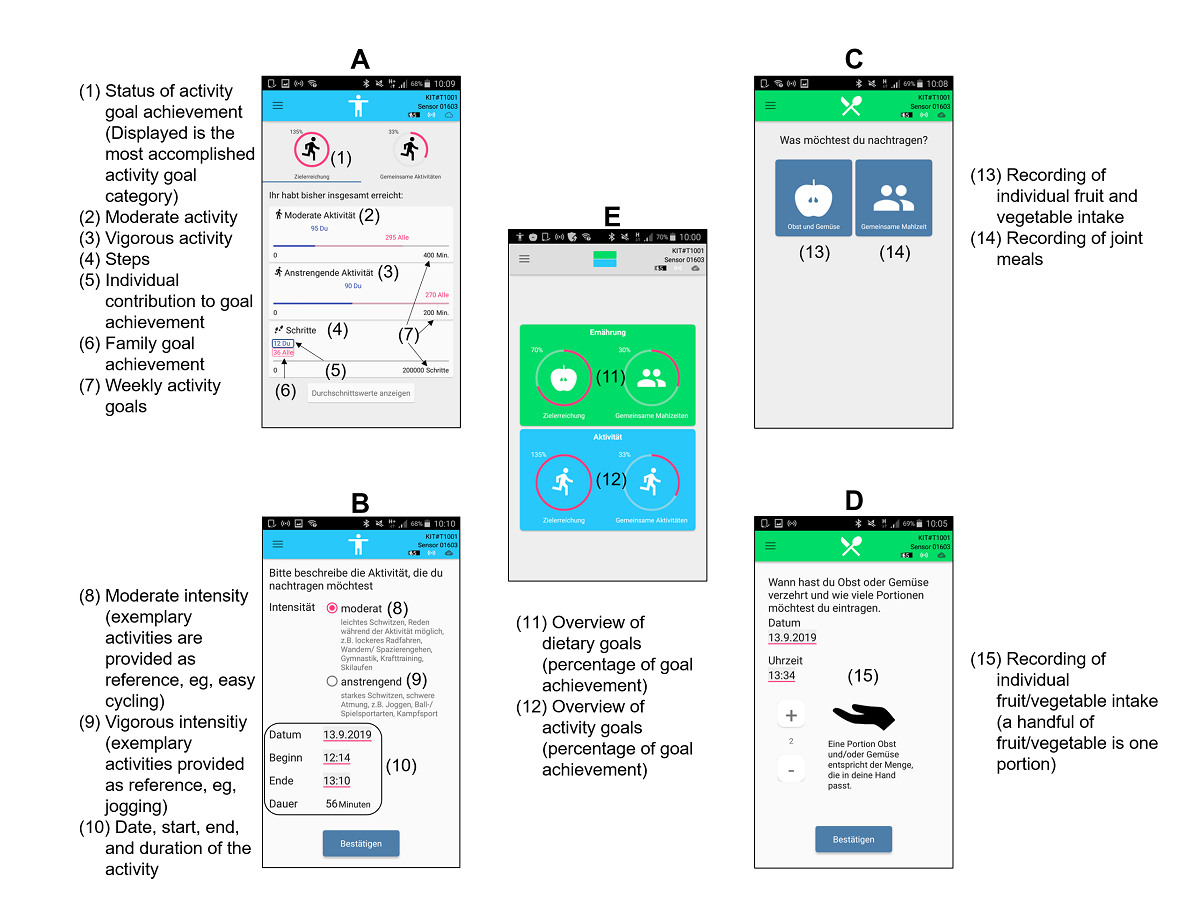
Examples of SMARTFAMILY app screens. A. Detailed status of activity goal achievement; B. Manual activity recording; C. Food recordings; D. Manual food recording; E. Start screen with overview of goal achievement.

Examples of the SF app screens are shown in Figure 4. On the home screen, the app always displays the whole family’s current status of goal achievement as well as individual family member’s contribution (Figure 4, #11-12). During the course of 1 week, if milestones of 25%, 50%, 75%, and finally 100% of goal achievement is reached by the family, every family member receives a congratulatory message and a motivational reinforcement, that is, “Great! You’ve reached 75% of your goal. Go on, you are making a good progress.” Moreover, the detailed goal achievements concerning moderate and vigorous physical activity and steps (Figure 4, #1-7) as well as fruit and vegetable intake (not displayed) on family and individual basis can be examined. The achieved values for specific days are also presented in the calendar function (not displayed). Although physical activity is recorded automatically by the accelerometer, fruit and vegetable intake has to be entered manually into the app (Figure 4, #15). In case of physical activity that is not assessed by the accelerometer because it cannot be worn (eg, swimming) or cannot be validly captured by the hip-worn accelerometer due to a lack of lower body movements (eg, upper body strength exercises or bicycling), the app incorporates a feature to manually enter the individual amount of time spent with moderate or vigorous activity (Figure 4, #8-10). As the family members’ smartphones are connected with each other (via internet) and each smartphone is connected with its accelerometer (via Bluetooth low energy), all family members receive real-time feedback on individual and family-level physical activity behavior with respect to steps, moderate and vigorous physical activity, as well as on self-reported fruit and vegetable intake. This allows for continuous self-monitoring of behavioral goals of a family.

### Features of the SF2.0 App

**Figure 5:**
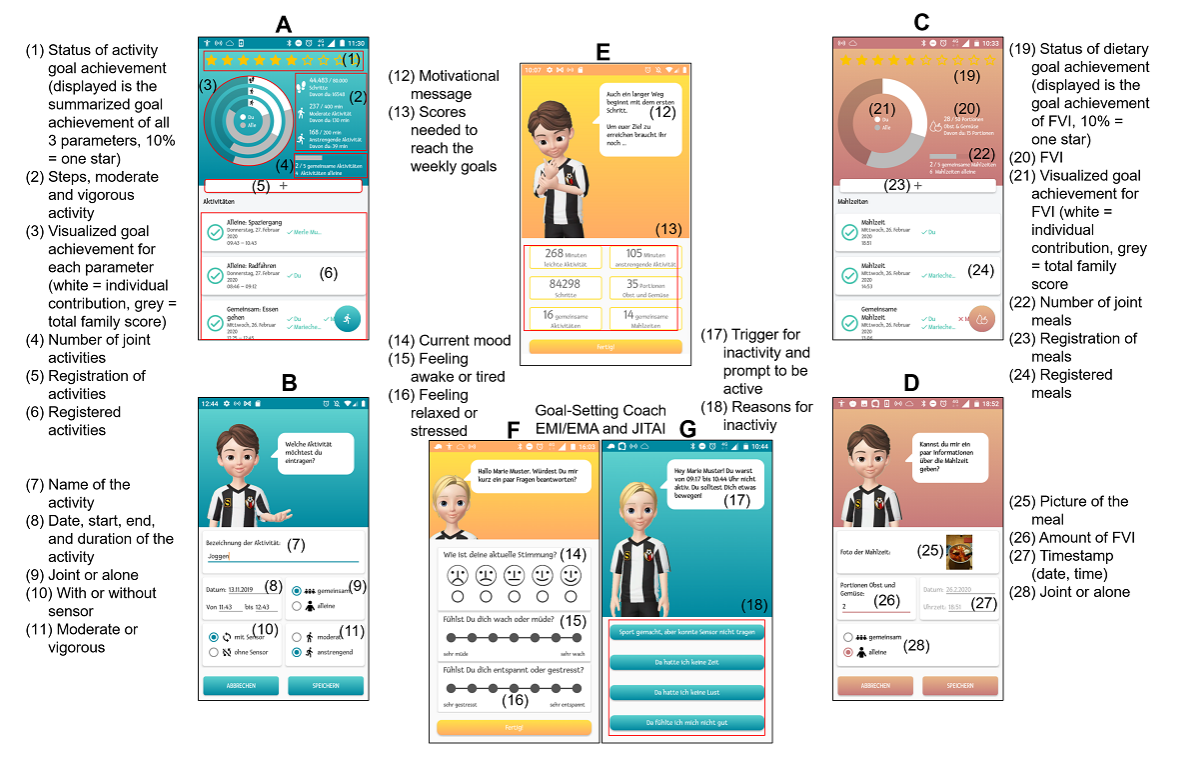
Examples of SMARTFAMILY2.0 app screens. A. Detailed status of activity goal achievement; B. Manual activity recording; C. Nutritional goal achievement; D. Manual food recording; E. Morning screen with an overview of the status quo and remaining physical activity and fruit and vegetable intake until goal achievement; F. Ecological momentary assessment and ecological momentary intervention; G. Just-in-time adaptive intervention. EMA: ecological momentary assessment; EMI: ecological momentary intervention; JITAI: just-in-time adaptive intervention; FVI: fruit and vegetable intake.

Table 1 provides an overview of the behavior change techniques incorporated in the SF app and the additional behavior change techniques of SF2.0 (Figure 5), allocated to the basic psychological needs [47].

Figure 5 shows examples of the SF2.0 app screens. While SF focuses on elementary functions, SF2.0 includes a more comprehensive range of app features such as JITAIs [58] and an interactive, humanized goal-setting coach who interacts with the app user in a personal way, providing hints and facts to achieve a higher app (and therefore, health behavior) commitment (ie, gamification). In both apps, triggered and app-based ecological momentary assessment (EMA) [59] is used. Supplementary to EMA of physical activity and healthy eating in SF, EMA was extended in SF2.0 by the real-time measurement of behavioral and affective correlates of physical activity and fruit and vegetable intake, including current mood, stress, and exhaustion (see #14-16 in Figure 5) as additional control variables [54,55]. This assessment is prompted at least 4 times a day, paralleling inactivity prompts (see below). Inactivity-triggered prompts are sent when the participant is inactive for at least 60 minutes (neither <2 sensor values at >2 MET nor 100 steps). Push notifications regarding inactivity are inhibited for the remaining day if the participant reaches at least 60 minutes of moderate-to-vigorous physical activity on the respective day. Every evening at 7 PM, participants are asked if they recorded all the necessary manual information of physical activity and healthy eating. The last assessment of mood, exhaustion, and stress occurs when the participant presses the “going-to-sleep button” (if there has not been an inactivity trigger during the last 60 minutes). Sleep quality is assessed every morning after the participant pushes the “get-up button” in the app. Furthermore, EMA is used to evaluate the reasons for inactivity (see #18 in Figure 5).

To further increase motivation, SF2.0 comprises a more detailed gradation of goal achievement. Participants can gather stars for every 10% of goal achievement. If the family achieves their individual goal during 1 intervention week, they are instructed to set a higher goal for the next week. In SF2.0, the interactive goal-setting coach advises them about a promising goal for the following week. If the family does not achieve its individual goal, the coach instructs family members to set the same or even a slightly lower goal for the next week. For recording of the different parameters and operation principles of SF2.0, see SF. One additional feature included in SF2.0 is adapted from the SMARTACT Toolbox [53-55]. Here, users are instructed to take a picture of every single meal (including snacks), producing an exact timestamp of food consumption (Figure 5, #25). Furthermore, SF2.0 comprises a real-time assessment of mood, exhaustion, and stress (Figure 5, #14-16), a gamification approach (visualized by the personal coach and collectable stars; Figure 5, #1), an inactivity-triggered ecological momentary intervention (Figure 5, #17,18), and a provision of up to 5 health-related facts by the interactive coach to improve health literacy [60]. These additional features are related to the inclusion of further behavior change techniques as shown in Table 1.

**Table 1:** Implementation of the basic psychological needs and behavior change techniques within the app features.

Self-determination theory, basic psychological needs	Autonomy	Relatedness	Competence
SF^a^ app features and number of behavior change techniques (Michie et al [37])	Self-imposed weekly goal-setting (eg, steps, duration of moderate-to-vigorous activity, fruit/vegetable intake) (behavioral goal-setting, #5^b^) App displays current status on performance and goal achievement (prompt review of behavioral goals, #10, prompt self-monitoring of behavior, #16, provide feedback on performance, #19) Calendar displays overview on performance (provide feedback on performance)	App implemented in a family-based setting, encouraging social support (plan social support/social change, #29) and identification as a role model (prompt identification as role model/position advocate, #30)	Set slightly higher weekly goals than current performance (set graded tasks, #9); rewards are provided according to progressively set goals via motivation notifications (shaping, #14) Motivation notifications at a level of 25%, 50%, 75%, and 100% of goal achievement (prompt rewards contingent on effort or progress toward behavior; provide rewards contingent on successful behavior, #12, #13)
Additional app features in SF2.0	Includes all the features of the SF app.	Additional review of the common goals via daily notifications in the morning (prompt review of behavioral goals, #10)	Interactive goal-setting coach provides knowledge about physical activity and healthy eating (provide information on consequences of behavior in general, #1) Gathering stars when achieving goals for each 10% of goal achievement (prompt rewards contingent on effort or progress toward behavior; provide rewards contingent on successful behavior, #12, #13) Inactivity-based reminders for physical activity according to ecological momentary intervention principles (teach to use prompts/cues, #23) Ecological momentary assessment of sleep, mood, exhaustion, and stress (barrier identification/problem solving, #8)

^a^SF: SMART*FAMILY*.

^b^These numbers refer to the Behavior Change Techniques (BCTs) shown in Michie et al [37].

### Outcomes

All self-reported measures and diaries are implemented in German language.

#### Primary Outcomes

Primary outcome measures are device-based measured physical activity (ie, amount of steps taken, minutes of moderate-to-vigorous physical activity) via accelerometers (T_0_ and T_1_) as well as self-reported assessed physical activity levels via questionnaires (T_0_-T_2_) and diary records (T_0_ and T_1_). Furthermore, healthy eating (fruit and vegetable intake) is assessed via questionnaires (T_0_-T_2_) and diary records (T_0_ and T_1_). Primary outcomes are similar for SF and SF2.0.

#### Device-Based Measured Physical Activity (Accelerometers)

Hip-worn accelerometers (Move 3/Move 4) are used to continuously record physical activity (3-axial acceleration, which is conveyed by algorithms into steps, time spent during moderate [3-6 MET]/vigorous [>6 MET] physical activity and sedentary time [1-1.5 MET]). The accelerometers are connected via Bluetooth low energy with the smartphone app to provide users direct feedback on their physical activity levels. The accelerometer’s validity was tested in previous studies and is considered accurate for assessing physical activity [61,62]. Participants are instructed to wear the accelerometer during wake time and remove it only for taking a shower, swimming, or during certain sports involving bodily contact to minimize the risk of injuries. This nonuse time is added manually into the app. Device-based activity assessment takes place at T_0_ and T_1_.

#### Self-reported Physical Activity (Questionnaire and Diary)

Self-reported physical activity is measured using valid and reliable measures. In the SF trial, the International Physical Activity Questionnaire [63] was used at all 3 measurement points for adults [64]. For children, the 60-minute screening measure for moderate-to-vigorous physical activity is used. These measures were chosen in order to account for the requirements of different age groups. However, due to better comparability of results, an adapted version (referring to the activities of the last week and not of a typical week) of the General Physical Activity Questionnaire [65] is used in SF2.0 for children and adults. In both trials, all participants complete a diary complementary to accelerometers [66], indicating time and type of activity, duration and intensity on each single day of the measurement week, and if this activity is carried out as a family or alone.

#### Fruit and Vegetable Intake (Questionnaire and Diary)

Fruit and vegetable intake is assessed using a single item asking for the total amount of fruits and vegetables consumed within a typical week [67] in the questionnaire as well as using a description in a diary of detailed food consumption during 1 week by indicating the time of the meal, ingredients, portions of fruit and vegetable intake, and whether the meal was consumed within the family or alone.

#### Secondary Outcomes

##### Demographics

In the T_0_ questionnaire, demographic information of the participants is collected, including sex, age, height, weight, highest education level, and tobacco and alcohol use (parents, only in SF), and attended school level (children). Moreover, participants are asked to rate their perceived general health [68]. The remaining questions are kept consistent over the 3 measurement points and are similar for SF and SF2.0.

##### Intrinsic Motivation Toward Physical Activity

To assess activity-related intrinsic motivation, the Behavioral Regulation in Exercise Questionnaire [69] is used [70].

##### Intrinsic Motivation Toward Healthy Eating

For assessing dietary-related intrinsic motivation, the Regulation of Eating Behavior Scale [71] is used.

##### Self-efficacy for Physical Activity and Healthy Eating

Activity-related self-efficacy and dietary-related self-efficacy are assessed using the health specific self-efficacy scale [72].

##### Family Health Climate

The family health climate is assessed using the family health climate scale [73].

#### Additional Outcome Measures of SF2.0

##### Intention to Participate

The intention to participate in physical activity and to eat healthy is assessed by a single-item measure [74,75]. Additionally, these measures were adapted to capture the participants’ intention to use smartphone apps for the promotion of physical activity and healthy eating.

##### Additional Measures in SF2.0

Healthy eating is assessed as fulfilment of the 10 guidelines of the German Nutrition Society [76] using the respective items of the Food Frequency Questionnaire [77,78] and diary information. Within the intervention, time and frequency of (shared) meals can be analyzed using timestamps of pictures taken [54]. Additionally, *adherence and user engagement* within the intervention are controlled for in SF2.0 by using app usage data stored on a server at the University of Konstanz (see Figure 2). Hence, app usage data (eg, recording of fruit and vegetable intake, achievement of physical activity and fruit and vegetable intake–related goals), and device-based measured physical activity are analyzed.

### Data Analysis

First, the baseline characteristics of the study population are summarized within each cluster randomized group on individual and family levels for all measures to control for group differences. Then, all primary outcome data will be screened for normal distribution by using the Shapiro-Wilk test. Data will be checked for outliers. To further analyze changes in health behavior, mixed-model analysis of covariance with time (T_0_ and T_1_ for device-based physical activity; T_0_, T_1_, and T_2_ for self-reported physical activity and healthy eating) as within-subjects and group (intervention group vs control group) as between-subjects factor will be conducted, with covariates being family health climate, self-efficacy, and intrinsic motivation (Behavioral Regulation in Exercise Questionnaire and Regulation of Eating Behavior Scale) as well as intention in SF2.0. Results of the Mauchly test will be checked for homoscedasticity of data and results will be corrected accordingly (ε>0.75 Huynh-Feldt, ε<0.75 Greenhouse-Geisser). Furthermore, homogeneity of the error variances will be checked, as assessed by Levene test. If this test does not reveal significance, a Box-Cox transformation will be applied to the data. Moreover, Tukey-corrected posthoc tests will be considered for detailed interpretation of results. Main effects will only be considered if the interaction is found to be significant. All analyses will be conducted using SPSS 26 statistical software (IBM Corp).

## Results

This study is funded by the German Federal Ministry of Education and Research and ethically approved by the Karlsruhe Institute of Technology. For both trials, it is hypothesized that the apps will positively influence physical activity and healthy eating in the whole family. Furthermore, SF2.0 is expected to produce stronger effects (ie, higher effect sizes) as compared to SF. SF app development and piloting are completed. Data acquisition for the SF trial is terminated and discontinued due to the COVID-19 pandemic. SF2.0 app development and piloting are completed, and data acquisition is ongoing. The recruitment of the participants for the SF2.0 trial started in February 2020. The results for SF are expected to be published in mid-2021, and the results of SF2.0 are expected for mid-2022.

## Discussion

### Overview

The aim of the SF project is the development, implementation, evaluation, and refinement of an mHealth intervention to increase physical activity and healthy eating at individual and family level. Extending the previous research, the behavior of children *and* parents is targeted in order to induce individual behavior changes that are anchored in daily family life. Moreover, several behavior change techniques were included, which contribute to the fulfillment of the basic psychological needs according to the self-determination theory [46,47]. We examined whether (1) mHealth interventions (SF, SF2.0) elicit meaningful increases in physical activity levels and healthy eating in children as well as adults as compared to controls (no mHealth intervention), (2) changes are maintained after the intervention period, and (3) intervention effects can be strengthened by the addition of app-based features and JITAIs (SF2.0).

### Innovative App Features: Strengths, Challenges, and Limitations

The SF and SF2.0 target the family as a whole. SF and SF2.0 aim to promote parents’ as well as children’s behavior by focusing on family level behavioral goals that could only be achieved if all family members collaborate. Although most apps that aim to improve physical activity and healthy eating focus on an individual’s behavior and comprise social features by the facilitation of social comparisons [79], both SF apps focus on collaborative group behavior and collective goal setting within a family-based setting. Furthermore, as known from sports psychological team theories, individual team members have the capacity to influence the behavior of other team members, thereby resulting in a state of team synergy, which can be loosely described as performance capacity that is more than the sum of its parts [80,81]. One advantage of using a group intervention is that studies have shown groups to work more effectively for a given goal (ie, aiming for a healthier lifestyle or increasing physical activity [82]). In one of the first studies in the field of social psychology, Triplett [83] found that people perform tasks better when the social context includes other people than when individuals complete a task alone. Subsequent findings validated Triplett’s results, and other experiments have shown that the presence of others can increase performance in many types of tasks, including jogging, playing pool, lifting weights, and working on mathematics and computer problems [84-86]. The tendency to perform tasks better or faster in the presence of others is known as social facilitation. This study aims to take the advantage of the social facilitation theory by involving the whole family as a social system into the intervention.

A constraining factor might be that family sizes and ages within families may vary. As studies have shown that there is an inverse relationship between family size, parental resources, and children’s educational performance [87], family size might also affect intervention success. However, there is currently a lack of knowledge about this relationship regarding behavior change or accomplishment of healthy lifestyles in families. Depending on family size distribution, families of different sizes will be compared if possible. However, future studies need to focus on and examine whether bigger families have advantages or disadvantages regarding intervention effects compared to smaller families. A further constriction might be the age range, especially of children and adolescents. Since this study includes the whole family, children of different ages and with different needs and perceptions are addressed in a similar way by the app, which might affect the intervention effects. Finally, SF and SF2.0 are based on sophisticated technical issues. EMA is used to assess physical activity (accelerometers), healthy eating (diaries), and psychosocial correlates. The inclusion of device-based and self-reported measures of physical activity provides a more comprehensive picture of the actual amount of physical activity. However, the synchronization of accelerometer-based data among multiple users also enhances the complexity of the app and is a potential source of problems caused by the Bluetooth low energy interface.

To the best of our knowledge, this is the first study implementing a mobile app to promote individual physical activity and healthy eating of children/adolescents and their parents in a family-based setting. Evidence-based strategies are integrated within a collaborative approach, which is characterized by setting family goals and collaborative striving for the achievement of these goals. This principle is contrary to several commercial apps or social media–based interventions, fostering a competitive environment through social comparisons among users. Further, the app in this study does not require external goal-setting but rather encourages the whole family to set their own goals and to plan joint activities and meals, which fosters communication within the family. Moreover, the additional inclusion of app features such as the interactive goal-setting coach, gamification, JITAIs, and EMAs in SF2.0 may exploit the potential of an mHealth intervention by means of its interactive and time-adapted nature [58,88]. Thus, JITAIs target to promote physical activity and healthy eating during those time periods when the individual is at high risk for physical inactivity and unhealthy eating patterns. Furthermore, user engagement will be monitored with these sophisticated tools, thus enabling the identification of the potential effects of regular app usage on the change of health habits.

### Potential Methodological Issues

Based on literature regarding theories on behavior change (ie, the transtheoretical model [89,90]), an intervention duration of 3 weeks might not be sufficient [35]. This might also be true for the follow-up at 4 weeks following the intervention, which might not be an appropriate time point to measure the maintenance of behavior change. However, mHealth intervention studies have revealed significant behavior change effects even with intervention durations of only 1 [91], 2 [92], and 3 weeks [93]. In a similar vein, a recent meta-analyses on mobile apps for diet management showed that interventions with longer duration were not generally more effective [35]. To our knowledge, there is currently no common accepted standard and sufficient empirical evidence for devising an “ideal” intervention duration, although a dose-response relationship appears very plausible. Moreover, as we examine families in their natural setting, there are also practical constraints. In Germany, a continuous school period lasts for a maximum of 6 to 8 weeks, followed by a vacation period. In order to conduct the core assessments, including pretesting and posttesting accelerometry, during 1 continuous school period, we needed to condense the actual interventions per family to 3 weeks. Longer intervention periods would inevitably mean that there is a confounding between assessment periods (school time vs vacation). Another issue is that participants have to use an additional smartphone to run the SF and SF2.0 app while also wearing the accelerometer on the hip. This burden might limit user engagement, which, however, can be controlled for by analysis of app usage data.

### Conclusion

Taken together, SF and SF2.0 expand on the existing body of evidence as they investigate the influence of a theory-based mHealth intervention targeting physical activity and healthy eating in a collective family-based setting. The major advantage of this smartphone app is that it facilitates behavior change at the individual level and the family level as the implemented strategies address changes in daily family life. Furthermore, motivation-enhancing features based on gamification strategies (ie, personal coach in SF2.0) and a JITAI approach matching interventions to individual needs is expected to induce positive behavioral changes at the individual and family level. Project information, updates, and results can be found on the project homepage [57].

## Appendix

Multimedia Appendix 1 CONSORT-eHEALTH checklist (V 1.6.1)

## Abbreviations

EMA: ecological momentary assessment
JITAI: just-in-time adaptive intervention
mHealth: mobile health
SF: SMARTFAMILY
